# A Laboratory Word Memory Test Analogue Differentiates Intentional Feigning from True Responding Using the P300 Event-Related Potential

**DOI:** 10.3390/brainsci9050109

**Published:** 2019-05-14

**Authors:** John F. Shelley-Tremblay, Joshua C. Eyer, Benjamin D. Hill

**Affiliations:** 1 Department of Psychology, University of South Alabama, Mobile, AL 36688, USA; bdhill@southalabama.edu; 2 Capstone College of Nursing, University of Alabama, Tuscaloosa, AL 35487, USA; joshua.c.eyer@ua.edu

**Keywords:** feigning, malingering, event-related potentials, clinical neuropsychology, P300

## Abstract

Symptom exaggeration and feigned cognitive impairment occur commonly in forensic and medicolegal evaluations. As a result, methods to detect feigned cognitive impairment are an indispensable component of neuropsychological assessments. This study reports the results of two neurophysiological experiments using a forced-choice recognition task built from the stimuli of the Word Memory Test and Medical Symptom Validity Test as well as a new linguistically informed stimulus set. Participant volunteers were instructed either to do their best or to feign cognitive impairment consistent with a mild traumatic brain injury while their brain activity was monitored using event-related potentials (ERP). Experiment 1 varied instructions across individuals, whereas Experiment 2 varied instructions within individuals. The target brain component was a positive deflection indicating stimulus recognition that occurs approximately 300 ms after exposure to a stimulus (i.e., the P300). Multimodal comparison (P300 amplitude to behavioral accuracy) allowed the detection of feigned cognitive impairment. Results indicate that, for correct responses, P300s were equivalent for the simulated malingering and good effort conditions. However, for incorrect responses, feigned impairment produced reliable but significantly reduced P300 amplitudes. Although the P300 is an automatic index of recognition—even when knowledge is hidden—its amplitude appears capable of modulation by feigning strategies. Implications of this finding are discussed for research and clinical applications.

## 1. Introduction

Exaggerated or feigned cognitive impairment is a significant impediment to obtaining valid clinical data, particularly in forensic and medicolegal contexts. Estimated base rates for malingered cognitive impairment range from 30–40% for medicolegal and neuropsychological evaluations [1,2], with even higher rates reported in disability evaluations [3]. The cost of malingering is high as feigned impairment is estimated to cost nearly $6 billion annually for worker’s compensation cases alone [4]. However, malingering diagnoses are difficult to substantiate as they require demonstrating both intentionality and motivation for an external reward, such as financial compensation [5]. A lower bar is used to identify those giving suboptimal effort, a term used to describe unsatisfactory performance due to inattention, poor motivation, exaggeration, or likely feigning. 

Expert consensus is that inattention, exaggerated or feigned impairment, and malingering occur on a continuum of effort [6,7]. Therefore, a substantial amount of research in clinical neuropsychology has focused on developing reliable methods for identifying suboptimal effort (SOE) with a high degree of specificity, where SOE ranges from intentional feigning to poor motivation. Current methods focus on either symptom validity, using implausible self-report, or performance validity, using highly atypical outcomes on tests that index actual abilities [8]. Symptom validity testing identifies feigning using suspect responses on self-report inventories such as the “Fake Bad” scale on the MMPI-II [9] or Structured Inventory of Malingered Symptomatology [10]. Performance validity testing identifies suspect patterns of performance on measures of actual cognitive ability, as stand-alone measures that only measure effort or embedded measures that are part of a more complete test of actual ability. Of the stand-alone measures, one of the most rigorously validated is the Word Memory Test [11]. 

While the technology to accurately detect feigned cognitive impairment has improved significantly in the last 20 years [12], the field still relies almost exclusively on either symptom self-report or behavioral responses to identify such individuals. A meta-analysis by Vickery and colleagues [13] found that sensitivity and specificity varied quite a bit across tests of performance validity and that many sacrifice sensitivity (ability to detect feigning) in order to maximize specificity (ability to reject feigning). Additionally, tests of performance validity are known to be vulnerable to coaching, a growing problem in medicolegal evaluations [14]. Coaching occurs when lawyers, support groups, or similarly motivated entities instruct an individual on how to circumvent performance validity tests for the purpose of receiving a financial compensation claim [15,16]. The WMT is resistant to coaching as it appears to be a standard clinical test and many consider it the gold standard for the detection of suboptimal effort using word stimuli [17,18,19], along with the Test of Memory and Malingering TOMM, [20,21], which is picture based. 

The most extensively validated performance validity test in clinical forensic settings [11], the WMT, exhibits excellent sensitivity and specificity due to its serial structure that features both repetitive and conceptually different tests assessing memory for a list of paired words. Nevertheless, new methods are needed that are more sensitive, reliable, and objective than current measures. One increasing area of focus is the addition of neuroscience to malingering detection, particularly neurophysiological methods, e.g., [22,23,24,25]. While these methods are not immune to coaching, the strategies necessary to defeat them are complex and can be countered by the psychologist administering the test [26,27].

Event-related potentials (ERPs) are a noninvasive, low-risk technique for measuring changes in brain functioning that uses electrodes placed on the scalp to collect electrical patterns of activity in cortical neurons. These continuous electroencephalographic recordings are then trimmed or epoched around each stimulus and averaged across trials of a specific type, increasing the signal-to-noise ratio for brain wave activity tied to that stimulus type. As a result of their ease of use and objective recording of cognitive activity, ERPs are a potentially important method to improve the sensitivity and diagnostic confidence of existing performance validity tests. For example, the integration of ERPs with the Guilty Knowledge Test (GKT) improved diagnostic accuracy [28,29], and more importantly, Vagnini and colleagues [24] reported basic evidence that ERPs have utility for discriminating simulated malingerers from patients with actual brain injury and accompanying cognitive impairment. More recently, these types of paradigms have been dubbed Concealed Information Tests (CITs), and have been shown to be very sensitive for the detection of concealed information when the P3 waveform is measured properly, from a negative to positive peak [26,27,30].

The primary ERP component used in deception detection is the P300, also called the P3, and it is best defined on the basis of (a) polarity (positive), (b) latency (which, however, is influenced by modality, endogenous processes (i.e., cognitive or elicited reactions to the properties of a stimulus). task requirements, and stimulus complexity and clarity), (c) morphology (it must have an identifiable peak), (d) scalp distribution (Pz and Cz > Fz, Pz usually > Cz), and, when possible, (e) well-established relationships with experimental manipulations (in particular probability and target effects) [31]. P3s are greatest when a participant must make a decision about a task based on the stimuli. P3 amplitude increases with task difficulty, the relative probability of the target stimulus, and the amount of effort the participant is putting into the task [32]. Generally, easy tasks with low probability target stimuli (*p* = 0.11 may be best) and good participant effort produce the greatest evoked P3 amplitudes [32,33]. Additionally, greater amplitude P3s may be elicited by a task-based, performance-contingent reward that is both valuable to the participant and strong (e.g., visible, large, expensive, etc., [34]). 

The two primary methods to elicit P3s are (1) as a reaction to the rarity and importance of the target stimulus (an “oddball” paradigm; [35], or (2) as a reaction to a contextually different and low-probability item that the participant has been primed to expect, e.g., a “mismatch” paradigm [36]. It is important to note that the P3 response to task-relevant information is an automatic, involuntary cognitive response that occurs before and independently of the participant’s behavioral response [37]. One consequence of this fact is that researchers have only recently examined differences between correct and incorrect responses in these studies [38]. This is germane because incorrect responses could signal either feigning or a true deficit in memory or effort.

Research that uses ERPs as a supplement to suboptimal effort detection is relatively rare, as the majority of this work deals with the detection of “guilty” or concealed knowledge within a forensic context [30]. However, van Hooff and colleagues [25] published a highly relevant test of ERPs used with a performance validity test. They employed a simulator paradigm with the Amsterdam Short-Term Memory (ASTM) test in a sample of 26 native English speakers, who were instructed to provide full effort or to feign a memory deficit (simulator group). The researchers found that ERPs were the same for both full effort and simulators at both early (300–500 ms) and late (500–800 ms) ERP periods, regardless of response accuracy. Yet, reaction times (RT) were significantly slower and more variable for simulators. They concluded that ERPs could be used to identify individuals who were feigning by comparing their behavioral errors with brain activity indicating a learned pairing, and, in their sample, a discriminant function analysis categorized full effort/simulators with 91% accuracy. 

The van Hooff study should be viewed cautiously, however. The analyses were based on only eight participants (full effort participants made few errors), their ERP results are unclear, and the researchers did not control for true forgetting. Despite low power, the researchers theorized their results as providing evidence for a conscious suppression mechanism that would activate when those in the simulator group chose either 1) to say “No” to a trial that they recognized, or 2) to say “Yes” to a trial that they did not recognize. Yet this strategy depends critically on brain differences between correct and incorrect trials: a difference that did not emerge in their data. These authors also did not control for the presence of trials that the simulators may have legitimately forgotten. One possible result of this would be that a reduced P3 amplitude associated with a genuine lack of recognition might have reduced the averaged amplitude for that condition. Consequently, there is a clear need for research on the utility of ERPs used in conjunction with symptom validity tests to detect feigning. Research in this area would benefit from investigating the role of correct and incorrect responding on a trial-by-trial, subject-by-subject basis, using a rigorous design and sufficient power to rectify the problems described previously. 

### Current Study

The present study was composed of two experiments. Experiment 1 was a between-subjects design using simulated malingerers who were given a new task that employed stimuli from the WMT and the similar but more brief Medical Symptom Validity Test (MSVT) [39]. These stimuli were combined and shown to participants in a study phase and then a test phase and these stimuli are referred to as the Modified Word List (MWL) task for Experiment 1. The primary purpose of this study was to determine whether the addition of ERP data to validated cutoff criteria for behavioral data could increase classification accuracy. This study served as a conceptual replication of the van Hooff study [25], with replacement of the ASTM with the more widely-used WMT and MSVT. The second experiment was conducted to replicate the first study using a new set of stimuli that were free of the experimental limitations of the WMT and the MSVT, primarily the lack of control of the degree of associative relatedness between the stimuli, which could easily confound any ERP results. The second experiment also employed a within-subjects design to control for intra-subject variability and to allow for the determination of exactly which stimuli were missed in the full effort condition. 

## 2. Experiment 1

### 2.1. Materials and Methods

#### 2.1.1. Participants

**Figure 1:**
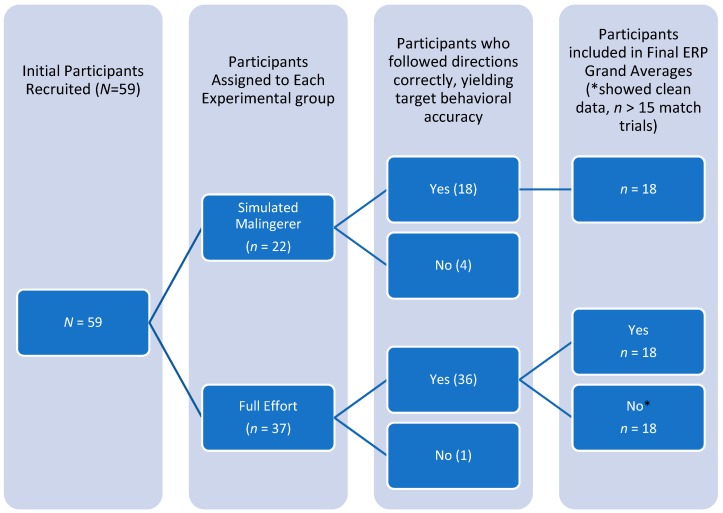
Study 1, Participant Recruitment Chart.

The sample was 36 undergraduate psychology students who were recruited through the university psychology department’s participant pools at two public, southeastern universities in the United States, and who received course credit for their participation. Entry criteria were that they be native speakers of English, right-handed, with normal-to-corrected vision, and free of neurological illness, psychoactive medication, or learning disability. They were assigned to either the Control condition, (*n* = 18; male = 6, female = 12) or to the experimental condition, hereafter referred to as Simulators (*n* = 18; male = 7, female = 11). The mean age for the control group was 23.9 years (SD = 7.9) and for the experimental group was 24.4 years (SD = 7.21). All participants were provided with study information and completed informed consent. Participant recruitment is outlined in Figure 1.

#### 2.1.2. Materials

##### Simulated Malingering Script

The experimental manipulation in this study was a script given to participants asked to simulate a brain injury that has been used in previous studies [40]. In the feigned condition, the undergraduates were given a scenario in which they pretended to be in a lawsuit after a car accident. They were given the following instructions: 

“In this experiment, we are going to be showing you some word pairs and you will then be asked to recall which words went together. You will be shown one word and then asked to choose which one of several words is its correct match. People who have suffered a brain injury have been known to have problems on these types of tasks. Brain injured people recall far fewer matches than do people whose brains are uninjured. Although you do not have a brain injury, for this experiment we would like for you to pretend that you do. Specifically, imagine that you have been in an accident in which you suffered a mild head injury or concussion. Because you were only slightly hurt, you will be able to do this task reasonably well, but not as well as an uninjured person. The better you are at making your test scores look like that of a person with a brain injury, the more money you might receive in a legal settlement. In this study, we will be awarding $20 gift cards to the best fakers. To win the award, you should try to answer in a way that will make it look like you have suffered a brain injury. Many brain-injured people will only get approximately 40% to 60% of the items correct. Keep in mind that too many errors will make it look like you are exaggerating your problems. This often results in it being easy for someone to catch your exaggeration. In the real world, this would result in a judge deciding that you are actually fine and deserve no money for your alleged injuries. In our experiment, it will mean you will unlikely be selected as one of the participants who will be awarded a $20 gift card. So, we would like you to do your best at simulating the performance of a brain-injured person. If you do this better than other study participants, you will be selected to win one of the prizes.” 

##### Word Memory Test

The Word Memory Test (WMT) [41] is a validated, stand-alone, performance validity test, composed of 20 simple semantically-linked word pairs. (Actual stimuli are confidential but follow the form of “butter–knife” or “dog–tail.”) Examinees are presented with three subtests: Immediate Recall (IR), Delayed Recall (DR), and Multiple-Choice (MC). Pairs are presented twice in learning trials for three seconds per presentation. Participants are tested on IR first, followed by DR 30 minlater using different distracter words. The IR testing task is a two-alternative, forced-choice task in which a participant must identify the target word pairing from a cue word, ignoring the distracter. The MC test presents a similar choice with 5 distractors. Each word pair is presented once in 40 choice-trials. Scores are percent recalled for each task and a Consistency (CON) score that indicates whether the participant missed the same words on the IR and DR subtests. The WMT has been extensively tested on actual, clinical samples. Participants’ scores were compared to the published cutoffs typically used for identifying suboptimal effort. Individuals with mild head injuries, traumatic brain injuries, and even children all scored well on this test for each of the three measures [42]. The WMT has high internal consistency and good test–retest reliability, and inter-correlations between the subtests are high (IR–DR = 0.94, IR–CON = 0.87, DR–CON = 0.88). 

##### Medical Symptom Validity Test

The Medical Symptoms Validity Test (MSVT) [39] is a simplified and brief version of the WMT, comprising 10 word pairs that are distinct from the WMT. These pairs are presented similarly to the WMT with two learning trials prior to IR and DR administrations with the only difference being that the DR is administered after 10–15 mininstead of 30 minfor the WMT. The IR and DR tasks are a two-alternative, forced-choice paradigm where the participant must indicate which of two words was previously learned, ignoring a simultaneously presented distracter word. There are 10 choices, one for each word of the pairs. Scoring and interpretation are similar to the WMT and the psychometric properties are also similar.

##### Modified Word List

For this ERP study, we combined the 20 word pairs from the WMT and the 10 word pairs from the MSVT, converting them to a digital presentation on a computer display. Stimuli were administered following the completion of the WMT and MSVT IR and DR subtests and the format was similar to the Multiple-Choice Recognition trial of the WMT that can be administered following the DR subtest. It was necessary to combine the stimuli to form the Modified Word List (MWL) recognition stimuli in order to yield an adequate number of experimental trials for ERP data processing. Permission for this modification and use was provided by Green’s Publishing, Edmonton. Any modified version of these tests (e.g., the MWL) remains copyright Green’s Publishing and Dr. Paul Green. During the task, participants were shown one word from the previously memorized pairs (the cue/base word) and asked to press a button when they saw the matching word belonging to the pair (the match/target word). Match/target words were located in a randomized position among the seven distracters (match–distractor probability = 0.13) and all words were presented sequentially with responses required for all eight words. Each word was presented for 1250 ms and centered horizontally and vertically on a black screen set to 640 × 480 resolution. To differentiate the base word from the other stimuli, it was presented first in all blue capital letters (medium blue, 26 pt. font). Subsequent words were displayed afterwards in the same position and font size with gray text. The inter-stimulus interval between words in a group was 1250 ms and was longer between the previous group of nine words and the new base word (2000 ms). The MWL displayed 270 total stimuli (30 word pairs from WMT and MSVT: target word first, followed by seven foils and the paired associate in a randomized order) using E-prime software [43]. The 26-point, bold text was displayed in black on a light blue screen to match the presentation of the WMT and MSVT. The cue/base word (displayed in medium blue) was shown for 1250 ms and indicated to the participant which word pair was targeted. The possible matches also appeared for 1250 ms with a 1950-ms interval and could have been the actual associate, a related lure (distracter) or a new foil. Participants were instructed to hit the “1” button if the word was a foil (an incorrect foil) or “2” button if it was a target (a correct paired associate).

### 2.2. Procedure

All participants provided informed consent, and all study procedures and materials were reviewed and approved by the Institutional Review Board. Following consent, participants were given instructions detailing each task. For those in the feigning simulator condition, a script of instructions directed them on how to feign the symptoms of a brain injury. Afterwards, all the participants received the learning trials of the WMT and MSVT followed by the IR subtests. During the subsequent break, participants were fitted with a 40-channel electrode cap. Researchers targeted consistent impedance values below 5 kOhms. Participants were then seated comfortably in a darkened room approximately 70 cm from a 19-inch, flat-screen monitor and completed the DR subtests of the WMT and MSVT. Afterwards, participants were given an opportunity to ask questions related to their expected and desired performance as well as specifics about the ERP aspects of the procedure. Next, the MWL was presented, and electrophysiological data were recorded as they responded for approximately 15 minusing a behavioral response box. The MWL comprised 270 total stimuli that were responded to while having ERPs recorded and essentially functioned as multiple-choice subtest found on the WMT. 

### 2.3. ERP Recording

Continuous EEG data were recorded from 38 sintered Ag/AgCl electrodes on the international 10–20 system. Bipolar HEOG and VEOG signals (horizontal and vertical eye channels) were recorded from the supra and infra-orbital areas, and the left and right canthi, respectively. Neuroscan NuAmps digitized the signal from DC to 300 Hz with a 60 Hz firmware notch filter at a rate of 500 Hz. Offline, 1000 ms epochs were formed by stimulus type, resulting in 601 samples per epoch. The epochs were screened manually for artifact, then all epochs with voltages exceeding ±60 μV (70 μV for four participants) were rejected. For participants with pervasive blink artifact, an ocular eye movement-reduction algorithm was applied before averaging of the waves [44]. Averages for each condition were created for each participant then averaged across participants. Total average accepted trials were 167.5/210 (79.8%) for the foils and 23.3/30 (77.8%) for the matches.

### 2.4. Data Analysis

A complex factorial ANOVA design was employed using accuracy, reaction time, and mean ERP amplitudes as dependent variables. The two within-subject variables were two levels each: stimulus type (match or foil) × accuracy (correct or incorrect); and the between-subjects factor was experimental condition (simulators vs. full effort). Visual analysis verified scalp distribution and the electrode site with maximal mean P3 amplitude. Behavioral responses that were outside ±2 standard deviations of the mean RT for each participant were excluded. ERP components were visually identified based on inspection of the grand average waveforms for each condition. For the P3 component, the peak deviation between the correct (C) and incorrect (IC) conditions was noted, and a 100-ms window was created centered on it. Mean amplitude measurements were computed using SCAN 4.3 software, Neuro Scan Labs, Sterling, VA, USA), which was entered into a repeated measures ANOVA. Mauchley’s test for sphericity was computed for within-participants factors, and Levine’s test of equality was computed for between-participants factors, with Greenhouse–Geisser corrections to degrees of freedom and *p*-value applied when necessary. Power reported is partial eta squared (η^2^) as given by SPSS V.22 (SPSS Inc., Chicago, IL, USA). 

### 2.5. Results

#### 2.5.1. Behavioral Reaction Time and Accuracy Data for the MWL Task

**Figure 2:**
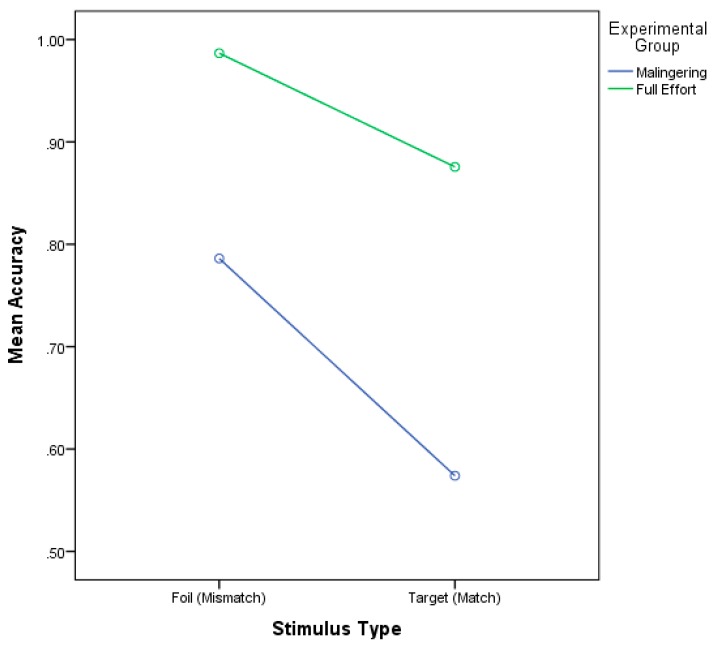
Mean accuracy percentage for each stimulus type by group.

Behavioral accuracy and reaction times were recorded during the administration of the ERP task. An initial review of the data showed a significant violation of homogenous variances for accuracy data in the mismatch condition, Levene’s test of equality (1,46) = 101.72, *p* < 0.001. Consequently, univariate results are reported using the Greenhouse–Geisser correction. The results of a 2 × 2 ANOVA using mean accuracy for Stimulus Type × Group produced a significant main effect for Stimulus Type, F (1,46) = 30.92, *p* < 0.001, η^2^ = 0.48, and for group, F (1,34) = 100.53, *p* < 0.001, η^2^ = 0.75, but not a significant interaction, *p* = 0.09. See Figure 2. For stimulus type, accuracies for the Match word condition (M = 72.5%, SE = 0.03) were significantly less than for the Mismatch condition ((M = 88.6%, SE = 0.02), Difference = 16.2%). For group, accuracies for the simulated malingering group (M = 68.0%, SE = 0.02) were significantly less than the full effort group ((M = 93.1, SE = 0.018); Difference = 25.1%). These data indicate that participants answered correctly more often for the mismatch words than the match and that, on average, the full effort group responded correctly to significantly more of the matching stimuli than the simulated malingering group. 

Reaction time data were reviewed, and all participants were removed for whom no data was available in the Incorrect-Match condition because of 100% accuracy (analysis sample: 10 Full Effort and 17 Simulator condition). The results of a 2 × 2 × 2 ANOVA using mean reaction time for stimulus Type × Accuracy × Group produced only a significant main effect of Type, F (1,25) = 4.55, *p* = 0.043 (See Table 1). Incorrect responses were slow across all categories. However, for the full effort category, incorrect responses were 68.41 ms slower, whereas for the simulators, incorrect responses were actually 6.36 seconds faster. This reinforces the notion that different strategies were in use by the two groups of participants consistent with group instructions to give full effort or feign impairment.

#### 2.5.2. ERP Results

**Figure 3:**
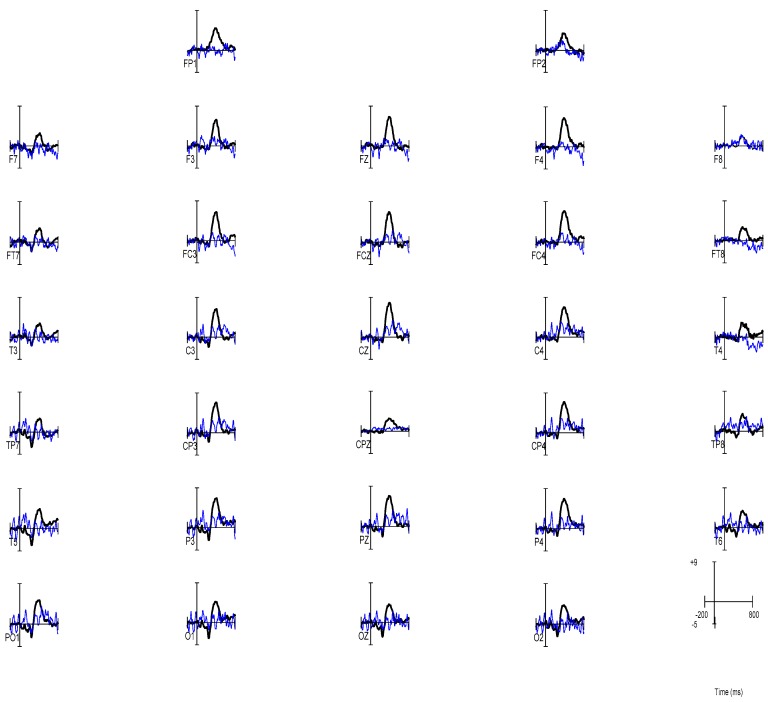
Grand average waveforms for the P3 subtraction wave (Match–Mismatch) for the Full Effort participants (black line) and the Malingering participants (blue line).

The first analysis performed on the ERP data was to identify the electrode location showing a maximal P3 amplitude, indicating recognition of the memorized paired associate. Notably, the initial investigation indicated that the measured effect was broadly distributed across the scalp. Measurement of the peak amplitudes of the grand-averaged waveforms revealed the presence of a P3 component with a maximum amplitude at Pz, (See Figure 3). Consistent with previous literature on the P3 component [45], all subsequent analyses used the Pz electrode site. The latency of the peak at this electrode was observed to be 410 ms for the simulator group and 382 ms for the good effort group. 

A 2 × 2 × 2 ANOVA was performed on the mean amplitudes of the P3 component at electrode Pz using the factors of Type (Match/Mismatch), Accuracy (Correct/Incorrect), and Group (Full Effort/Simulated Malingering) producing a significant main effect of Type, (F (1,34) = 21.67, *p* < 0.001, η^2^ = 1.00), a marginal effect of Accuracy (F (1,36) = 3.90, *p* = 0.057, η^2^ = 0.10), and an interaction between Type and Accuracy (F (1,34) = 16.22, *p* < 0.001, η^2^ = 0.97). The 3-way interaction between Type, Accuracy, and Group was also marginal (F (1,34) = 4.00, *p* < 0.054, η^2^ = 0.49). To explore these interactions, data for the simulated malingering and full effort conditions were reviewed separately. See Table 2 for descriptive data for each condition.

**Figure 4:**
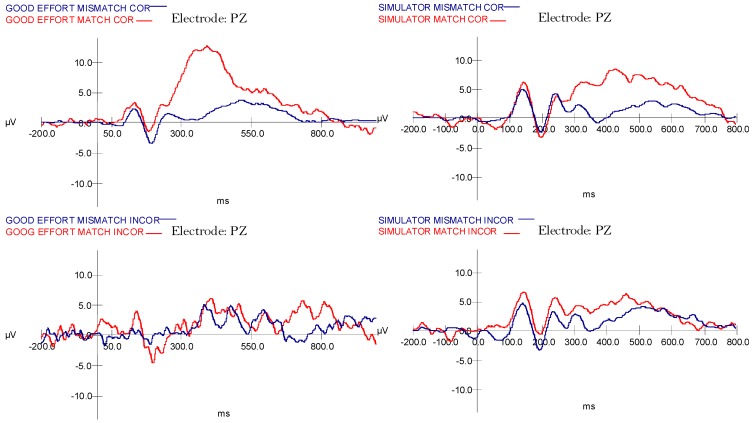
Grand average waveforms for P3 for Malingering and Full Effort groups, showing the effects of behavioral response accuracy.

In the Full Effort condition, a 2 (stimulus type) × 2 (accuracy) ANOVA using mean peak amplitude at the Pz electrode showed a significant main effect of Stimulus Type (F (1,17) = 5.44, *p* = 0.03, η^2^ = 0.24 (MMismatch = 2.67, SEMismatch = 0.99; MMatch = 6.54, SEMatch = 1.81)), a marginal effect of Accuracy (F (1,17) = 3.97, *p* = 0.06, η^2^ = 0.19 (MCorrect = 4.62, SECorrect = 0.92; MIncorrect = −1.22, SEIncorrect = 2.17)), and a significant interaction between Type and Accuracy (F (1,17) = 11.83, *p* = 0.003, η^2^ = 0.41). As shown in Figure 4 (right side), the effect of accuracy on Pz amplitude was significantly greater in the Match condition than the Mismatch. To investigate this interaction, we separated the analyses by Accuracy (See Figure 4). For Correct trials, results indicated that Pz mean peak amplitude was significantly higher for the Match stimuli than for Mismatch, F (1,17) = 64.40, *p* < 0.001, η^2^ = 0.791 (MMismatch = 1.79, SEMismatch = 0.69; MMatch = 11.16, SEMatch = 1.57), but mean peak amplitudes did not differ for Incorrect stimuli, F (1,17) = 0.28, *p* = 0.601, η^2^ = 0.016 (MMismatch = 3.55, SEMismatch = 1.80; MMatch = 1.93, SEMatch = 2.89).

For participants in the Simulated Malingering condition, a 2 (stimulus type) × 2 (accuracy) ANOVA showed only a significant main effect for Stimulus Type, where the Match condition showed a stronger P3 than the Mismatch condition (F (1,17) = 29.96, *p* < 0.001, η^2^ = 0.64 (MMismatch = 1.17, SEMismatch = 0.86; MMatch = 6.08, SEMatch = 1.21)). There were no other significant effects. However, the interaction between Stimulus Type and Accuracy showed a medium-to-large effect size and a trend verging on significance, prompting further exploration (F (1,17) = 4.42, *p* = 0.051, η^2^ = 0.21). This pattern of means was approximately the same as for the Full Effort condition, where Pz mean peak amplitude was higher for the Correct Match condition than other conditions. However, the topography of the P3 was different and showed a less distinct, lower amplitude waveform.

### 2.6. Discussion: Experiment 1

The results of Experiment 1 provide compelling information for the proposition that the P300 can be used to detect intentional feigning of cognitive impairment. Consistent with the literature on ERPs and memory, we found distinctive patterns of P3 responses that differed by task instructions. For those instructed to give good effort, the Correct Match condition elicited a large mean amplitude at P3 that was not present for Incorrect or Mismatch stimuli (See Figure 4, top). This activity indicated that their greatest recognition response occurred for trials where the target stimulus was followed by a correct response representing accurate recall. In contrast, the group instructed to simulate malingering had a different pattern. As shown in Figure 4, simulators exhibited an identifiable but smaller P3 response for Correct Match stimuli. This indicated that while participants were performing the same task on each trial, the neural processing underlying simulated malingering were likely different. Additionally, for the full effort category, incorrect responses were 68.41 ms slower, whereas for the simulators, incorrect responses were actually 6.36 seconds faster indicating that simulators feigning impairment made quick decisions to choose incorrect responses relative to genuine errors by individuals giving good effort. 

A central assumption of this study was that a substantial P3 would be statistically significant for the participants in the Simulator condition, thereby serving as in index of true recognition regardless of behavioral response. These results, however, indicate that the P3 cannot be thought of as a simple indicator of recognition memory. If that were true, the P3 should have been as large in the Simulator condition as in the Full Effort condition. Instead, P3 was clearly more robust in individuals given good effort and less robust in individuals feigning impairment for actual recognition trials indicating that a cognitive response set can moderate P3. However, P3 was still present and recognizable in individuals feigning impairment, supporting that it has utility as a neurophysiological index of recognition validity. This differential pattern of P3 elicitation supports the use of P3s in comparison with behavioral responses as a promising method of identifying deceptive responses.

Several factors may explain the reduction in P3 amplitude during simulated malingering. The first is that missed trials were actually due to a failure of memory. If this were the case, the P3 recognition response would not occur, and thus the averaged P3 would consist of a mixture of intentional incorrect answers (measurable P3) and true failures of memory (small or no P3) that would combine to yield a reduced amplitude grand average. Supporting this proposition, a low amplitude deflection that did not achieve statistical significance was noted. Another possibility is that individuals cognitively suppress the word representations of the correct responses in order to facilitate the generation of a false response. Supporting this proposition, the observed waveform for Simulators in the Correct Match condition differed notably in topography from the waveform for Correct Waveforms. 

Experiment 1 concluded that Simulated Malingering could be identified using ERP waveforms, but weaknesses were noted. First, it did not determine the extent to which the waveform was influenced by forgetting vs. intentional suppression or both. Second, the relative number of Match stimuli were too limited. And third, the word stimuli may be strengthened to provide more reliable test stimuli. 

## 3. Experiment 2

A second experiment extended the investigation of Simulated Malingering using the P300 by addressing several weaknesses from the Experiment 1. In order to investigate the issue of true forgetting versus intentional suppression, a within-subjects design was implemented where all participants performed the task at Full Effort and then repeated the task using a Simulated Malingerer strategy. Through this design, unlearned pairs from the Full Effort condition can be removed from analyses in the Simulated Malingering condition. While it is possible that participants could forget the stimuli between conditions, it is unlikely as the Full Effort condition would in reality serve as another opportunity to view and strengthen the memory pairings. The average delay between conditions was only approximately 15 minutes.

A second issue was the relative lack of trials available for the formation of grand averages. For the critical Match targets, there were only 30 stimuli presented, leaving even fewer trials when incorrect trials are removed. In Experiment 1, subjects were instructed to aim for 50% accuracy, which resulted on average in 14 correct trials/subject and 10 incorrect trials/subject. Thus, it is critical to have a larger number of stimuli to promote a better signal-to-noise ratio for the ERPs.

A final concern was that the stimuli from the WMT and MSVT, despite their proven clinical utility, were not specifically designed for cognitive research to control for associative strength between the target/base word and its match. Therefore, the degree of semantic relatedness was variable and sometimes poor. In ERPs, associative strength can modulate the amplitude of the N400 component which overlaps the P300 in time and space, confounding its interpretation [46,47,48] Thus, this research would benefit from a new word-pair list for use with ERPs that responds to these concerns. Ideally, it would be analogous to the WMT and MSVT word pairs but employ normed words and control for length and associate-associate strength for word pairs. 

Thus, Experiment 2 sought to determine whether the reduction in P3 amplitude observed in Experiment 1 was due to a failure of memory (honest forgetting) or the utilization of a conscious strategy on the part of the subject (suppression). It employs a new word-list task (Revised Modified Word List (RMWL), created and evaluated with naïve participants from the undergraduate participant pool using a within-subjects design. It was hypothesized that, even after the removal of trials that were not correctly responded to in the full effort condition, the incorrect trials would show a significant reduction in P3 amplitude compared to the correct trials in the simulator condition.

### 3.1. Materials and Methods

#### 3.1.1. Participants

The sample contained 18 (11 male, 7 female) undergraduate students who were recruited through a university psychology department’s participant pool and received credit for their participation. All the participants were instructed to complete both the full effort and simulated malingering experimental tasks. From this group, 3 participants were rejected because of excessive somnolence, incompliance with instructions, equipment failure, and/or high artifact. The final sample of 15 (9 men, 6 women) had a mean age of 20.5 (SD = 2.04). All participants gave informed consent, were native speakers of English, with normal-to-corrected vision and were free of neurological illness, psychotropic medication, or learning disability.

#### 3.1.2. Materials

##### Revised Modified Word List

The new task consisted of 350 stimuli using 50 word pairs that were chosen using the University of South Florida Free Association Norms (USFFAN) [49]. The first words (cue/base) were all common nouns or adjectives, and the associate (match/target) words were chosen as the most frequent associate from the USFFAN norms (See Appendix A Table A1). These words were also common, but included nouns, verbs, and adjectives. Five distracter/foil words per trial were chosen that were of the same approximate length as the match/target, but did not appear as associates on the USFFAN. The chosen number of stimuli per trial was based on the reported optimal probability for producing a P3 component of *p* = 0.11 [33]. The mean length of the cue/base words was 5.2 letters, 4.7 letters for the match/target, and 5.4 letters for the distracter/foil. The mean forward associative strength between the cue/base and match/target was 0.36.

#### 3.1.3. Procedures

During the task, the participants were shown one word from the previously memorized pairs (the base word) and asked to press a button when they see the matching word belonging to the pair (the match word). This word was displayed in a randomized position among the seven distracters. Presentation of words was one at a time and sequentially, similar to the MWL in Experiment 1, and functioned as an analog of the WMT Multiple Choice Recognition trial [41]. All 8 words (1 match and 7 foils) were presented and a response was required for each word. Each word was presented centered horizontally and vertically on a light blue screen. To differentiate the base word from the other stimuli, it was presented first in all blue, capital letters (medium blue, 24 pt. font). Subsequent words are displayed afterwards in the same position and font size with black text. There is a 1250-ms interval between any two stimuli. There was a 2000-ms interstimulus interval between the previous group of 7 words and the new base word. Subjects proceeded through each set 7 of words without a break using a different random set order for each participant.

### 3.2. Results

#### 3.2.1. Behavioral Reaction Time and Accuracy Data for the Revised Modified Word List

During the administration of the ERP task, behavioral accuracy was recorded and subsequently analyzed. An initial review of the data showed a significant violation of homogenous variances for accuracy data in the mismatch condition, Levene’s Test of Equality (1,24) = 32.01, *p* < 0.01. Consequently, univariate results are reported using the Greenhouse–Geisser correction. The results of a 2 × 2 ANOVA using mean accuracy for stimulus type (Foil vs. Match) × instruction set (Good Effort vs. Simulated Malingering) produced a significant main effect for stimulus type, F (1,14) = 29.00, *p* < 0.001, but not instruction set. There was a significant interaction between stimulus type and instruction set, F (1,14) = 8.01, *p* = 0.013 (see Table 3). The patterns of means indicated that mismatch stimuli were answered correctly more often than match stimuli and that the impact of task instruction was much greater for the match stimuli than the foil stimuli. Specifically, when compared with Simulated Malingering, Good Effort participants answered many more match stimuli correctly but equivalent numbers of foil stimuli.

The purpose of this analysis was to determine whether the instruction set influenced participant reaction time (RT). Incorrect trials were excluded because there were insufficient trials on the Good Effort condition to complete a valid analysis across conditions. Thus, a 2 (Instruction Set) × 2 (Stimulus Type) ANOVA was performed on the Mean RT data for all participants. The analysis yielded no main effect of Instruction Set (*p* > 0.05), a significant effect of Stimulus Type, F (1,14) = 19.3, *p* < 0.001, and no interaction (see Table 3). Thus, support was provided for the idea that instruction set had no effect on the participants’ reaction time in the presence of a significant overall effect.

To examine the impact of Simulated Malingering on reaction time, the next ANOVA investigated the effect on RT of the Behavioral Response Strategy (Correct vs. Incorrect) and Stimulus Type (Foil vs. Match). A significant main effect for Behavioral Response was obtained, F (1,14) = 10.07, *p* = 0.007, but not for Stimulus Type (see Table 4). Additionally, and most critically, a significant interaction occurred between Behavioral Response and Stimulus Type, F (1,14) = 12.05, *p* = 0.004. The pattern of means, shown in Table 5 below, demonstrate the impact of Behavioral Response strategy on Stimulus Type. For match trials, Response Strategy had almost no effect; however, for Foil trials, participants responded fastest when answering correctly and slowest when answering incorrectly. For correctly responded to trials, it is faster to say “No” to a foil than to say “Yes” to a match, and for the incorrectly responded to trials (simulator) it is faster to say “No” to a match than to say “Yes” to a foil.

#### 3.2.2. ERP Results

**Figure 5:**
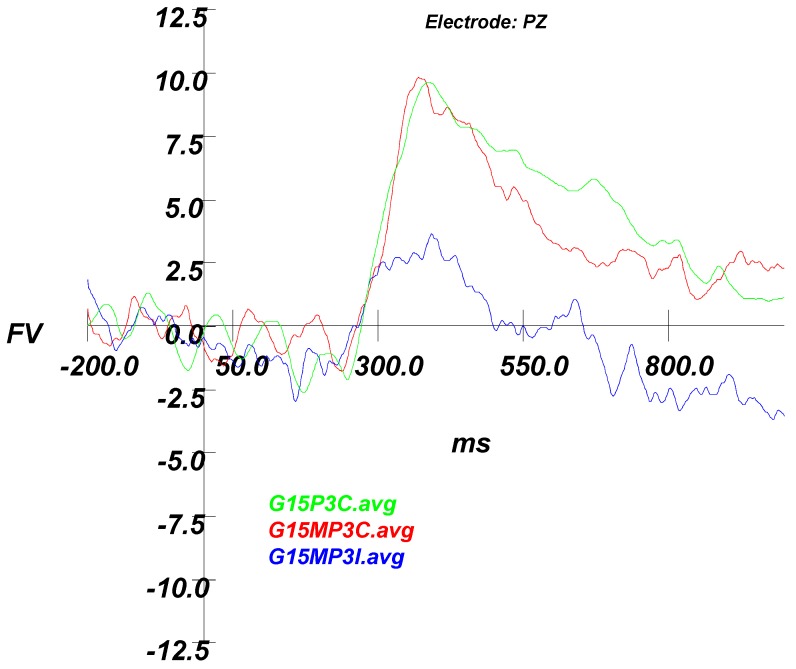
Experiment 2 grand average waveforms for Full Effort correct trials (Green) are not different from Malingering correct trials (Red), which are both different from Malingering incorrect trials (Blue). G15P3C = Grand Average Full Effort P3 Correct; G15MP3C = Grand Average Simulating P3; CorrectG15MP3I = Grand Average Simulating P3 Incorrect.

ERPs were formed in the same manner as Experiment 1, with the largest P300 peak still measured at the PZ electrode. Broadly, correct responses yielded large amplitude differences between foil and match stimuli; however, since the number of incorrect Good Effort trials were negligible (very few misses), no valid interpretation of this P3 was possible. Consequently, using only Correct trials, a 2 (Instruction Set) × 2 (Stimulus Type) ANOVA compared mean amplitude at Pz. The analysis yielded no main effect for Instruction Set (Good Effort vs. Simulated Malingering), F (1,14) = 0.021, *p* = 0.886 (see Figure 5). As expected, the effect of Stimulus Type (Foil vs. Match) was significant, F (1,14) = 55.718, *p* < 0.001, but not the interaction, indicating a significant P3 that was not modulated by instruction set. Since the amplitudes of the good effort and simulator correct condition trials was equivalent, the remaining analyses focused on the simulator condition.

**Figure 6:**
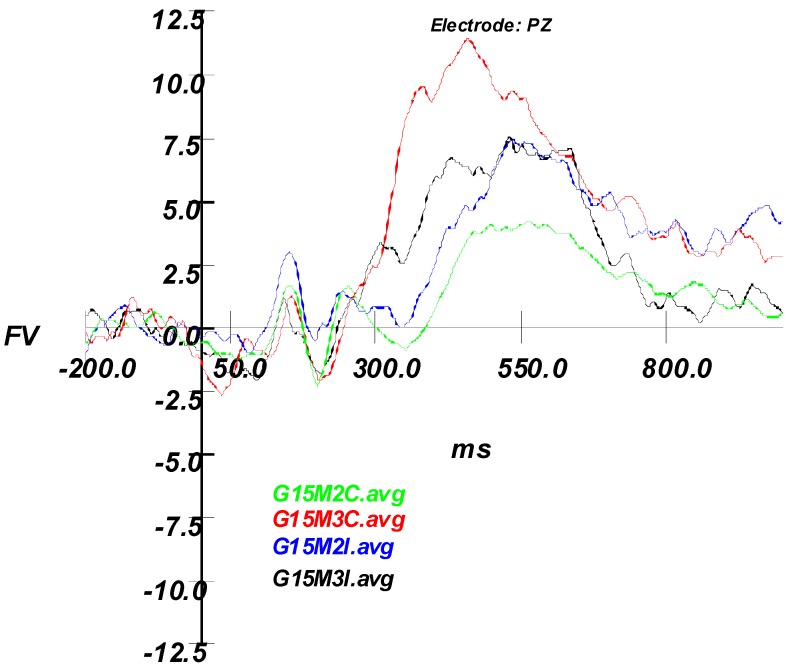
Experiment 2 grand average waveforms for all Simulating conditions. Note how the incorrect Match condition results in a smaller P3 response than the correct Match condition. G15M2C = Grand Average Simulating Mismatch Correct; G15M3C = Grand Average Simulating Match Correct; G15M2I = Grand Average Simulating Mismatch Incorrect; G15M3I = Grand Average Simulating Match Incorrect.

The final analysis was a 2 (Stimulus Type) × 2 (Accuracy) ANOVA on mean peak amplitude at the Pz electrode in the Simulated Malingering condition. Results indicated a significant main effect for stimulus type, F (1,14) = 22.730, *p* < 0.001, η^2^ = 0.619, but not for Accuracy, *p* < 0.15. This indicated that, regardless of accuracy, a significant P3 effect was found. Furthermore, and most critically, an interaction between stimulus type and accuracy emerged, F (1,14) = 12.28, *p* < 0.004, η^2^ = 0.467, providing support for the hypothesis that intentionally missed trials would result in lower P3 amplitudes (Figure 6).

### 3.3. Discussion: Experiment 2

Experiment 2 corrected several methodological issues associated with the stimuli used in Experiment 1 and followed up on hypotheses generated from the results of Experiment 1. As expected, the data provide evidence supporting the hypothesis that intentionally missed trials would result in lower P300 amplitude. Distinctive patterns of P300 responses were found which differed in the simulated malingering and good effort conditions. The mean amplitudes for all experimental conditions at electrode PZ showed that correct responses yielded larger amplitudes differences between foil and match stimuli. 

First, at the behavioral level, for correctly responded to trials, it was faster to say “No” to a foil than to say “Yes” to a match, and for the incorrectly responded to trials (simulator) it was faster to say “No” to a match than to say “Yes” to a foil. Thus, it is apparent that the decision to respond “No, that is not a correct match,” is faster than the decision to respond “Yes, that is a correct match,” regardless of whether the individual is feigning or giving good effort. This may be due to the ability of the semantic memory system to rapidly bias the episodic memory judgment. By constraining the size of the memory set search to a relatively small size, the decision to say no is made quickly, on the basis of poor semantic fit. Conversely, “Yes” responses may require an additional search of memory space to determine episodic familiarity prior to the decision point.

Regarding the ERP data, a comparison between the correct trials for good effort and simulator conditions showed significance in the effect of stimulus type (foil vs. match). This indicated a significant P300 that was not eliminated but somewhat reduced by instruction set. For the simulator condition, the results indicated that regardless of accuracy a significant P300 was found. Most importantly, the interaction between stimulus type and accuracy emerged, demonstrating that the participant’s behavioral accuracy instructional set to feign or give good effort was a significant mediator of the P300 response. This provided support that P300 elicitation can be used in comparison with behavioral responses as a powerful method of confirming feigned responses. This finding also points to the importance of looking at participant accuracy on a trial-by-trial basis as opposed to simply by instruction set.

## 4. General Discussion

Experiment 1 demonstrated that individuals giving good effort and individuals feigning cognitive impairment or simulating malingering produce similar P3 profiles for correct responses but there were different P3 profiles for incorrect responses. Specifically, a trend was observed where simulators seem to have a suppression of P3 when intentionally choosing incorrect responses that is distinct from individuals giving good effort who make genuine mistakes. While this trend approached significance for Experiment 1, the results of Experiment 2 replicate this observation providing supportive evidence of the reliability of this P3 suppression effect. Most critically, Experiment 2 employed more and better controlled word stimuli and demonstrated a statistically significant difference between correct and incorrect trials in the simulated malingering condition. There was also an interesting behavioral effect noted that “No” responses were generally faster than “Yes” responses regardless of whether the participant was giving good effort or feigning impairment on the forced-choice paradigm that was used in these studies and is common for PVT measures. Consistent with clinical lore that individuals feigning cognitive impairment respond more slowly than individuals giving good effort, we found that our simulators had overall longer reaction times to stimuli than our genuine effort group. However, we also found a specific pattern for simulators on incorrect responses where “No” incorrect responses were faster than “Yes” incorrect responses. This suggests that research examining slowed reaction time as a marker for feigned cognitive impairment needs to consider the interaction of the type and category of response being timed in these studies, particularly when algorithms are being derived from simple reaction time data.

The current study adds to the literature in several areas. First, we have demonstrated that both the original task of Green^41^ and an analogous task based on close semantic-associates yields a P300 event-related potential that can be useful for differentiating correct from incorrect trials in a simulated malingering paradigm. Our study also strengthens the conclusions of Van Hooff and colleagues [25] who suggested the operation of an “conscious suppression mechanism” that would be used to control the conflict between a participant’s true knowledge and their plan for executing a feigned response. The van Hooff and colleagues experiment hypothesized no ERP differences between their full effort and simulator conditions because of their assumption that the P300 (their early window) would be incentive to the participants task strategy on a trail by trial basis. For methodological reasons, they combined correct and incorrect trials for their analyses of between group differences. By adding our Experiment 2 with both more trials and an explicit statement of a 50% accuracy target, we were able to yield adequate trials to analyze simulator and full effort conditions independently for correct and incorrect trials. Van Hooff and colleagues took their lack of a significant difference between correct and incorrect trials as evidence that “the mistakes made by the simulators were not genuine but fabricated.” We agree with their conclusion, but for different reasons. By using a within-subjects design in Experiment 2, where we were able to explicitly control for the effect of true forgetting, we were able to independently isolate the ERP effect of intentional forgetting, and it is consentient with the operation of a conflict suppression mechanism.

Our study largely replicates the finding of Rosenfield, Ellwanger and colleagues [33,45] who showed that the combination of behavioral accuracy and ERP amplitude could be used to determine the presence of simulated cognitive impairment. However, our results go counter to those of Hoover, Zottoli, and Gross-Fifer [22] who found no differences in the auditory P3a response of simulators versus control participants. Their study employed behavioral measures of executive function as well as an ERP task but did not require participants explicitly to simulate during their ERP task, nor did they analyze correct trials separately from incorrect trials. Indeed, the critical question for these types of paradigms in how does a subject plan and executive a feigning trial? Our experiments suggest that when experimenters can control for true forgetting and analyze correct and incorrect trials separately there is reason to be believe that the P300 is not task invariant and may provide clues about the feigning techniques employed by the participants. Indeed, most recently, Rosenfeld and colleagues [38] have shown not only that P300 amplitude varies reliably for different conditions, but that additional ERP markers, such as the P900, can provide information about task-related strategies. 

There were some significant limitations to this study. Primarily, the use of malingering stimulators feigning impairment instead of known malingerers presents several problems. While this remains unproven, there is a possibility that the performance of undergraduate simulators differs from the performance of actual malingerers who are more likely to be highly motivated. Actual malingerers in litigation are more likely to have prepared or been coached by professionals for a neuropsychological assessment [50]. Also, it is unclear to what extent our participants successfully followed all the scripted malingering instructions. We do know that they maintained behavioral accuracy between 57% and 78%, on average, indicating that they were mindful of the instructions, “Many brain-injured people will only get approximately 40% to 60% of the items correct. Keep in mind that too many errors will make it look like you are exaggerating your problems.” If they had adopted a random strategy, their accuracy would have varied much more greatly. However, our findings still have significant applicability to the developing literature examining ERPs and deception and we believe the Experiment 2 presented here is stronger than previously published studies because of the use of normed word stimuli that allow for a control of the effect of associative strength on the subsequent ERP waveforms. Additionally, we demonstrate the potential utility of P300 for differentiating valid from feigned responding regardless of whether simulators would perform differently from known malingerers. 

## 5. Conclusions

The presented studies showed that there is a place for the continued development of ERPs for use with neuropsychological testing. In particular, these data support the finding that these two methods of assessment supply different types of information about an examinee’s responses on the tests and that P3 profile interpretation may have promise as a complimentary tool for behavioral measures of feigned cognitive impairment. Additionally, we report a novel interaction effect for reaction time and type of behavioral response that is specific for individuals simulating malingering. Consequently, the possibility remains for future research to streamline and economize this process into a highly useful experimental method for widespread employment in neuropsychological and clinical applications. With improvement, this combinatory method of detecting deception could potentially become a fixture in malingering assessment.

Cognitive data and reaction times can be used to identify feigned cognitive impairment. Cognitive event-related potential data showed that P300 profiles for correct responses were similar between those giving good effort and those feigning cognitive impairment but different for incorrect responses. Data showed a suppressed P300 response for incorrect responses in feigned cognitive impairment compared to genuine incorrect responses in the good effort condition. Reaction time data showed that individuals respond faster to “No” than “Yes,” regardless of whether they are feigning impairment or not, and that those feigning impairment tended to have longer reaction times than those who did, particularly for incorrect versus correct trials. 

## Figures and Tables

**Table 1:** The Effect of Experimental Group and Stimulus Type on Mean RT in Experiment 1.

Experimental Group by Accuracy by Type				
Experimental Group	Accuracy	Type	Mean	Standard Error
Simulators	Correct	Match	685.74	27.11
		Mismatch	725.50	26.94
		Total	705.62	
	Incorrect	Match	695.08	64.98
		Mismatch	703.43	40.60
		Total	699.25	
Good Effort	Correct	Match	599.57	35.35
		Mismatch	656.10	35.13
		Total	627.84	
	Incorrect	Match	656.71	84.72
		Mismatch	735.80	52.93
		Total	696.25	
		Total Match	527.42	
		Total Mismatch	564.17	

**Table 2:** Effect of Group, Stimulus Type, and Accuracy on Mean P3 Amplitude at Pz.

Group	Type	Accuracy	Mean	Standard Error
Simulators	Mismatch	Correct	0.52	0.69
		Incorrect	1.82	1.51
	Match	Correct	7.28	1.45
		Incorrect	4.89	2.36
Good Effort	Mismatch	Correct	1.79	0.69
		Incorrect	3.55	1.51
	Match	Correct	11.16	1.45
		Incorrect	1.93	2.36

**Table 3:** Group Comparison of Mean Accuracy for Experiment 2 (*N* = 15).

Condition	Mean (%)
MATCH STIMULI	
Simulated Malingering	56.67
Good Effort	73.13
Total	64.90
FOIL STIMULI	
Simulated Malingering	83.67
Good Effort	85.53
Total	84.60
Simulated Malingering	70.17
Good Effort	79.93
All Trials	75.05

**Table 4:** Effect of Response Strategy on Reaction Times for the Simulated Malingering Condition in Experiment 2 (*N* = 15) grouped by Accuracy.

Stimulus Group	Mean (ms)
CORRECT	
FOIL	563.08
MATCH	628.06
Total	595.57
INCORRECT	
FOIL	679.61
MATCH	625.60
Total	652.60
COMBINED	
Correct	595.57
Incorrect	652.60
All Trials	624.09

**Table 5:** Effect of Response Strategy and Stimulus Type on Reaction Times for the Simulated Malingering Condition in Experiment 2 (*N* = 15) grouped by Stimulus Type.

Stimulus Group	Mean (ms)
MATCH	
Correct	628.06
Incorrect	625.60
Total	626.83
FOIL	
Correct	563.08
Incorrect	679.61
Total	621.34
COMBINED	
Correct	595.57
Incorrect	652.60
All Trials	624.09

## Appendix A

**Table A1:** Complete list of Stimuli for Experiment 2.

Trial	Cue	D1	D2	D3	D4	D5	Match
1	dill	vanish	skill	reduce	shoulder	hourglass	pickle
2	speaker	bridge	food	French	garden	empty	radio
3	flex	bottom	deodorant	shock	drama	think	muscle
4	dam	bagel	roast	alike	booth	Egypt	water
5	peak	phony	dove	angry	dolphin	boiled	top
6	raspberry	harsh	poison	skin	dollar	judge	fruit
7	sleep	owner	snow	sink	injury	skirt	dream
8	hockey	tool	farm	sailing	poet	vagrant	puck
9	fuel	rebel	ice	candy	hostage	stir	gas
10	ruler	leaves	flush	chair	murder	rocky	measure
11	cricket	jail	member	pirate	rowing	tragedy	bug
12	paint	scribble	release	cheese	dresser	picture	brush
13	hotdog	cruel	fight	bed	disable	envelope	bun
14	cabinet	coed	donkey	camel	camp	David	kitchen
15	lion	garnet	kiss	shirt	dust	screen	tiger
16	dragon	coil	dodger	boring	plumber	magazine	fire
17	elf	button	gone	hatch	endless	Denmark	Santa
18	leopard	class	book	permit	tower	dump	spots
19	smoke	collar	curtail	escargot	snickers	slither	cigarette
20	pail	cup	pig	hedge	rocker	puncture	bucket
21	key	battle	oil	Casio	motion	rude	lock
22	cafe	plant	Indian	end	pistol	tall	coffee
23	plane	life	decide	partition	cell	note	fly
24	galaxy	fat	sonic	chick	add	spoon	stars
25	razor	stingray	bean	sore	pilsner	sun	sharp
26	virus	quest	glasses	action	welcome	resident	sick
27	volcano	forget	monkey	wrong	raid	mantis	erupt
28	king	pander	peanut	double	toast	sing	queen
29	vampire	cover	faucet	drive	hippie	condition	blood
30	soccer	bulb	plug	coke	vulture	diet	ball
31	goat	point	reach	forth	planet	begin	milk
32	corn	screw	foot	swallow	rope	electricity	cob
33	package	saliva	shovel	scream	ants	jury	box
34	tax	criminal	loser	brief	simple	lazy	money
35	cabin	doughnut	T.V.	wire	desk	driver	log
36	mug	ash	animal	bag	form	alive	beer
37	jazz	pilot	hermit	college	expired	alone	music
38	hatchet	boulder	sloppy	coat	cold	snooty	axe
39	rooster	dirt	tie	bang	bones	basin	hen
40	done	job	sky	space	glow	zipper	finish
41	town	dig	power	slander	should	train	city
42	tack	static	stiff	vacate	dance	cut	nail
43	swamp	digest	surgeon	circle	nice	tooth	mud
44	cavern	fan	white	high	slow	orange	cave
45	mustard	should	ugly	alien	lungs	confused	ketchup
46	ocean	busy	bathroom	jump	eggs	debate	sea
47	cereal	doll	file	long	sign	coins	breakfast
48	waffles	shake	wiggle	meek	despair	promise	syrup
49	cyclone	tulip	equinox	bike	exercise	wizard	tornado
50	nerd	salad	bacon	throat	friendly	cigar	geek

D = Distractor.
